# Soil functional responses to drought under range‐expanding and native plant communities

**DOI:** 10.1111/1365-2435.13453

**Published:** 2019-09-12

**Authors:** Marta Manrubia, Wim H. van der Putten, Carolin Weser, Freddy C. ten Hooven, Henk Martens, E. Pernilla Brinkman, Stefan Geisen, Kelly S. Ramirez, G. F. (Ciska) Veen

**Affiliations:** ^1^ Department of Terrestrial Ecology Netherlands Institute of Ecology (NIOO‐KNAW) Wageningen The Netherlands; ^2^ Laboratory of Nematology Wageningen University Wageningen The Netherlands; ^3^ Department of Soil Quality Wageningen University Wageningen The Netherlands

**Keywords:** litter mass loss, plant range expansion, saprophytic soil fungi, soil functioning, soil rewetting, summer drought

## Abstract

1. Current climate warming enables plant species and soil organisms to expand their range to higher latitudes and altitudes. At the same time, climate change increases the incidence of extreme weather events such as drought. While it is expected that plants and soil organisms originating from the south are better able to cope with drought, little is known about the consequences of their range shifts on soil functioning under drought events.
2. Here, we test how range‐expanding plant species and soil communities may influence soil functioning under drought. We performed a full‐factorial outdoor mesocosm experiment with plant communities of range expanders or related natives, with soil inocula from the novel or the original range, with or without summer drought. We measured litter decomposition, carbon mineralization and enzyme activities, substrate‐induced respiration and the relative abundance of soil saprophytic fungi immediately after drought and at 6 and 12 weeks after rewetting.
3. Drought decreased all soil functions regardless of plant and soil origin except one; soil respiration was less reduced in soils of range‐expanding plant communities, suggesting stronger resistance to drought. After rewetting, soil functioning responses depended on plant and soil origin. Soils of native plant communities with a history of drought had more litter mass loss and higher relative abundance of saprophytic fungi than soils without drought and soils of range expanders. Functions of soil from range expanders recovered in a more conservative manner than soils of natives, as litter mass loss did not exceed the control rates. At the end of the experiment, after rewetting, most soil functions in mesocosms with drought history did not differ anymore from the control.
4. We conclude that functional consequences of range‐expanding plants and soil biota may interact with effects of drought and that these effects are most prominent during the first weeks after rewetting of the soil.

A free http://onlinelibrary.wiley.com/doi/10.1111/1365-2435.13453/suppinfo can be found within the Supporting Information of this article.

## Graphical abstract

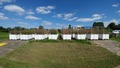

## 1 INTRODUCTION

Climate change enables latitudinal and altitudinal range shifts of species from warm to previously colder climate zones (Chen, Hill, Ohlemuller, Roy, & Thomas, 2011; Parmesan & Yohe, 2003; Pauli et al., 2012). This intracontinental migration makes that previously colder ecosystems are colonized by an increasing number of thermophilic plant species originating from lower latitudes, which is likely to be favoured by mild winter temperatures (Tamis, Zelfde, Der Meijden, & De Haes, 2005). As plant species live in association with above and belowground organisms (De Deyn & Van der Putten, 2005; Wardle et al., 2004), and many soil organisms are more limited in their dispersal ability than many plant species, plants and their associated soil organisms will colonize the new range asynchronously (Berg et al., 2010). Furthermore, it is increasingly acknowledged that although bacteria and fungi are widely present in terrestrial ecosystems, their diversity and composition follow global biogeographical patterns (Delgado‐Baquerizo et al., 2018; Fierer & Jackson, 2006; Tedersoo et al., 2014). As a result, in the new range, migrating plants may encounter a novel soil community, which may lead to novel plant–soil interactions (Berg et al., 2010; van der Putten, 2012). Thus far, very few studies have tested possible functional consequences of such asynchronous dispersal.

Plant range expansion may result in the release from plant‐specific root pathogens, which can favour the performance of range‐expanding plant species in their new habitat (Engelkes et al., 2008; Morriën, van der Putten, & Wurzburger, 2013). The establishment of range‐expanding plants may also alter pools and rates of carbon and nutrient cycling in the soil via the quantity and quality of the resources provided to the saprophytic community (Meisner, Boer, Cornelissen, & Putten, 2012). In a study that included range‐expanding plants from both intra‐ and intercontinental origin, range‐expanding plant species turned out to have a more unique chemistry than natives (Macel, Vos, Jansen, Putten, & Dam, 2014). Differences in plant chemistry are likely to influence the decomposability of the litter and thereby the soil carbon and nutrient balance. For example, invasive exotic plant species have been shown to alter both soil microbial community composition and pools and fluxes of carbon and nutrients in the invaded ecosystems (Ehrenfeld, 2010). However, the functional consequences of intracontinental plant range expansions on such ecosystem processes are unknown.

The ecosystems where range‐expanding plant species establish are also subjected to other effects of climate change, such as altered temperature and precipitation regimes and extreme weather events. For example, in the Netherlands and other parts of north‐western and central Europe, the severity and frequency of drought events is projected to increase with current climate change (EEA, 2016; KNMI, 2015). Drought events can directly affect soil microbial communities and important ecosystem functions such as primary productivity, decomposition and nutrient cycling, both at the scale of ecosystems (Setälä & McLean, 2004; de Vries et al., 2018, 2012) and of continents (Ciais et al., 2005). With ongoing intracontinental range expansions, effects of drought on ecosystem properties and processes will interact with effects caused by changes in plant and soil community composition (Bardgett & van der Putten, 2014; Classen et al., 2015). Although plant–soil interactions and drought stress are known to affect carbon and nitrogen cycling separately, the consequences of their interactive effects on soil functioning remain poorly understood (Bardgett, Manning, Morriën, & Vries, 2013; Kardol, Cregger, Campany, & Classen, 2010; Sanaullah, Blagodatskaya, Chabbi, Rumpel, & Kuzyakov, 2011). Combining these interactive effects of extreme drought events and plant communities of different origin in experiments is crucial for understanding the consequences of plant range shifts (Meisner, Deyn, Boer, & Putten, 2013) and exotic plant invasions (Alba, NeSmith, Fahey, Angelini, & Flory, 2017; Caldeira et al., 2015) on ecosystem functioning.

Range expansion of both plants and soil organisms may modify the impact of drought on ecosystem functioning. This is because the responses of soil microbial processes (e.g. respiration, enzyme activity) to current drought vary in their type and magnitude depending on historical climatic conditions (Averill, Waring, & Hawkes, 2016; Hawkes & Keitt, 2015; Hawkes, Waring, Rocca, & Kivlin, 2017). For example, soil communities from southern Europe that expand their range have evolved in regions with warm and dry conditions (e.g. Mediterranean and continental climates), which could affect their response to drought differently from communities that have evolved under more cool and wet high‐latitude conditions (e.g. Atlantic climate) (http://worldclim.org/). As a result, soil microbial communities originating from dry climate regions are generally more resistant to drought than communities from mesic climate (Manzoni, Schimel, & Porporato, 2012). Therefore, the effects of drought on soil functioning could be alleviated not only by the presence of range‐expanding plants but also by the presence of their associated southern soil communities. However, little is known about responses of ecosystem processes in soil to extreme drought events under the influence of range‐expanding plants with or without their own soil community.

The aim of our study was to assess the effects of range‐expanding plants and soil biota alone and in combination on soil functional responses to an extreme summer drought, as is predicted to occur more frequently in north‐western Europe under future climate conditions (IPCC, 2014). We focused on some soil functions that support ecosystem productivity and nutrient cycling, such as litter decomposition, soil respiration and extracellular enzyme activity. We also determined the relative abundance of saprophytic soil fungi. We measured all variables immediately after drought and at different times upon rewetting to determine the ability of the soil communities to withstand drought (i.e. resistance) and the rate at which communities are able to recover (i.e. resilience) (Allison & Martiny, 2008; Griffiths & Philippot, 2013; Pimm, 1984). We tested the hypotheses that (1) soils conditioned by communities of range‐expanding plant species are more resistant to drought than soils conditioned by plant communities of related natives and (2) soils conditioned by communities of range expanders recover faster after a drought event than soils influenced by communities of related natives. For both hypotheses 1 and 2, we expected that these effects would be stronger when range‐expanding plant species were grown in soils with a southern than with a northern soil community. To test our hypotheses, we set up a multiyear outdoor mesocosm experiment with communities of either range‐expanding or related native plant species growing in soils with or without a soil inoculum from the original range of the range‐expanding species, that is south‐eastern Europe. Two years after the set‐up of the outdoor mesocosms, we simulated a summer drought in half of the mesocosms. In our experiment, we focus on processes controlling the release of carbon from the soil (e.g. litter mass loss, respiration), but we did not determine C inputs into the soil.

## 2 MATERIALS AND METHODS

### 2.1 Mesocosm set‐up

In 2013 mesocosms of approximately 1 m^3^ were set up in the experimental garden of the Netherlands Institute of Ecology (Wageningen, the Netherlands). A total of 40 mesocosms were distributed in five rows of eight mesocosms with a spacing of 0.5 m between them (Figure S1). Mesocosms were filled with bulk soil collected from a riparian area in Boven‐Leeuwen, the Netherlands (51°53'56.80", 5°33'45.49"). The area of soil collection represents the natural habitat type of the plants chosen.

We inoculated the topsoil (~20 cm) of the mesocosms with 20% of field soil inoculum originating from a riparian area in the Netherlands, the expansion range (hereafter, northern soil), or south‐east Europe, where the range‐expanding plants selected are native (hereafter, southern soil). Northern soil was collected from the Millingerwaard natural area in the Netherlands (51°51'56.97", 5°59'33.60"). Southern soil was collected from a floodplain area near Solt in Hungary (46°47'58.95", 18°57'30.97"). Inoculation soils were collected from five independent locations in the field (minimum 60 m apart) and were kept separately to act as the five experimental replicates (blocks) in this experiment. With this inoculation method, we aimed to establish different soil communities while minimizing possible the abiotic soil properties, although with a 20% inoculum differences in soil abiotic properties cannot be completely ruled out as having influenced the results. In order to further reduce the possibility of changing abiotic soil properties by inoculation, we chose to collect field samples in riverine ecosystems that all receive soil from the same parent material (Central European Alps). Nevertheless, we cannot entirely exclude the possibility that there were some differences between the abiotic properties of the soil inocula from North and South. Prior to the start of the experiment, the composition of soil fungal communities was significantly different between the soils inoculated with the northern and southern inocula (Figure S2).

Thereafter, all mesocosms were planted with plant communities of riverine areas. In 2013 and 2014, we grew a community of three native grasses planted with either three native or three range‐expanding forbs to allow establishment of the soil inocula by conditioning the soil with a naturally occurring plant community. From 2015 onwards, we planted communities formed by range‐expanding and native forbs. We focused on forbs, because there are very little documented range‐expanding grasses in our study site (NDFF, 2018). We chose plant species that are currently expanding their range within Europe and paired them with species that belong to the same genus while being native in the Netherlands. Some range‐expanding species did not have a congeneric native species in the expansion range; for those species, we chose native plant species with similar traits and life strategies as a comparison. Selecting congeneric pairs of plant species that within pairs have similar functional traits allowed us to control for trait variation between the native and range‐expanding species as much as possible and hence to study the effect of plant origin (native vs. range expander) on soil processes (Manrubia, Snoek, Weser, Veen, & Putten, 2019). A complete overview of selected range expanders and related native species used can be found in Table S1.

All seeds were collected from field sites in the Netherlands. We collected seeds for the range expanders ourselves, while seeds of the native species were collected by an external supplier (Cruydt Hoeck). All seeds were germinated on sterile glass beads and then transplanted to trays with sterilized soil from the same origin as the background soil in the mesocosms. Trays were placed in the greenhouse until seedlings were approximately 10 cm in height; then, seedlings were transplanted to the mesocosms. In 2015–2016, we planted a total of 64 plant individuals (8 individuals of each plant species) following a regular squared 8 × 8 grid to ensure balanced plant communities in terms of diversity and evenness of species. Each year at the beginning of the spring, we re‐planted annual plants and perennial plants that had died off and removed weedy plant species.

### 2.2 Experimental design

In summer 2016, we installed rain shelters above all mesocosms and the drought treatment started resulting in the final full‐factorial experimental design with three factors and 40 experimental units: 2 water levels (control/drought) × 2 plant community types (native/range expander) × 2 soil inocula (northern/southern) × 5 replicates (blocks). Half of the mesocosms (control) were artificially watered with tap water two times a week to ensure a rainfall regime representative of the seasonal average precipitation in the area of the last five years (34 L/week, source: KNMI). The remaining half of the mesocosms received no artificial watering during a period of 6 weeks in order to mimic an extreme summer drought event (from June 28 to August 9). When the drought phase ended, rain shelters where removed from all mesocosms and we artificially watered the mesocosms when needed to ensure a minimum water input of 34 L/week for 12 weeks, which is when the last measurements were collected. In total, the experimental period lasted for 18 weeks and was divided in three phases of 6 weeks each: the experimental drought phase, an early recovery (rewetting phase) and a late recovery phase.

### 2.3 Measurements

#### 2.3.1 Litter decomposition

Litter mass loss was measured at the end of the three different phases of the experiment using a modified version of the Tea Bag Index method (Keuskamp, Dingemans, Lehtinen, Sarneel, & Hefting, 2013), a standardized protocol to assess decomposition of substrates of contrasting chemical complexity. Instead of a 3‐month incubation proposed by Keuskamp et al. (2013), we buried tea bags in the soil for a total of 6 weeks corresponding to our experimental phases. At the start of each period of 6 weeks, we buried pre‐weighed green and rooibos tea bags at approximately 8 cm below the soil surface in each mesocosm to match the depth at which soil sampling took place (0–10 cm). In this way, the chemical composition of the substrates was standardized at the beginning of each phase. The three sets of tea bags were buried in three locations in the centre of the mesocosms avoiding potential edge effects. In each of the mesocosms, plants were planted following a regular grid and tea bags were consistently buried beneath the same plant species in either native or range‐expanding plant communities. At the end of each 6‐week phase, we retrieved the teabags from the soil, removed big fragments of roots, oven‐dried the remaining tea material (70°C for 48 hr), dry sieved it to 0.4 mm to remove soil particles and weighed it to determine mass loss.

**Figure 1:**
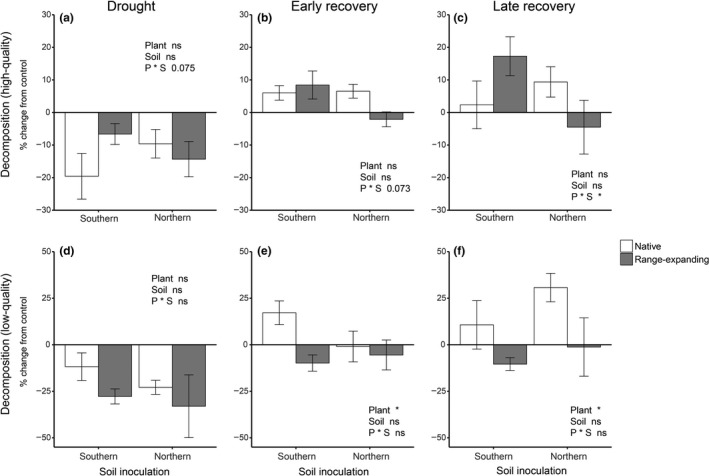
Proportional change from the control in mass loss of high‐quality substrate (a–c) and low‐quality substrate (d–f) during each of the 6‐week experimental phases: drought (a, d), early recovery (b, e) and late recovery (c, f) after drought. Bars indicate means with standard error (*n* = 5)

#### 2.3.2 Soil sampling

At the end of each of the 6‐week experimental phases (end of the drought period, 6 and 12 weeks after the end of the drought), we collected soil samples. We took a composite soil sample of four different soil cores (2.5 cm diam.; 0–10 cm depth) to obtain a representative soil sample from each mesocosm. Soil samples were immediately sieved through a 4‐mm sieve. We used a 4‐mm mesh instead of a smaller size to facilitate sample processing given that our soils did not contain small stones. Then, a subsample was collected in an Eppendorf tube, immediately frozen in liquid nitrogen and kept at −80°C for molecular analyses. The rest of the soil was kept at 4°C and darkness until further analyses, which were performed within a week after soil collection.

#### 2.3.3 Background environmental parameters

To determine how drought affected the content of nitrogen in the mesocosms, we quantified mineral nitrogen in the soil before the drought event started and at the end of the drought. We extracted soil available nitrogen (${\text{NO}}_{3}^{-}$, ${\text{NO}}_{2}^{-}$ and ${\text{NH}}_{4}^{+}$) from all mesocosm by shaking a 10 g dry weight equivalent in a 50 ml of 1 M KCl solution for 2 hr. We determined the concentration of mineral nitrogen using an autoanalyser (QuAAtro Autoanalyzer, SEAL Analytical Ltd.).

We measured plant biomass in November 2016, corresponding to the end of the growing season, to assess plant productivity under the different experimental treatments (Figure S3). For aboveground biomass, we subsampled a fixed row of eight individual plants per mesocosm containing one individual of each species. For root biomass, we collected three soil cores of 73.6 cm^3^ each (15 cm depth × 2.5 cm diameter) and subsequently washed the root material contained in each core to have an estimate of standing root biomass in the topsoil.

#### 2.3.4 Soil carbon mineralization and substrate‐induced respiration (SIR)

We measured soil carbon mineralization (basal soil respiration) in all soil samples. We weighed the equivalent of 4 g of dry soil in 50‐ml centrifuge tubes with a modified lid equipped with a rubber septum and a rubber o‐ring in order to ensure air tightness. Tubes were capped tightly, and we then flushed the headspace air in the tubes with CO_2_‐free air for 2 min at 1 bar (Westfalen Gassen Nederland BV). We incubated all tubes at 20°C for 24 hr in a climate‐controlled chamber (Economic Lux chamber, Snijders Labs). Subsequently, we took a 6.2‐ml sample of headspace air from each tube using a needle and stored it in pre‐evacuated air‐tight vial (Labco Exetainer). We determined the concentrations of CO_2_ in the gas vials (over pressure of 1 bar) by injecting 250 µl of each sample in a Trace Ultra GC gas chromatograph equipped with a flame ionization detector with methanizer (mFID) (Interscience BV) and a TriplusRSH autosampler (Interscience BV) and a Rt‐QBOND (30 m, 0.32 mm ID) capillary column (Restek). We used helium 5.0 as a carrier gas, a sample split ratio of 1:20 and set oven temperature at 50°C with a flow of 5 ml per minute. We used a calibration curve of known concentrations of CO_2_ ranging from 0 to 4,600 ppm of CO_2_ prepared from a reference gas (2.38% CO_2_ in synthetic air, Westfalen AG) to determine the amount of CO_2_ in our samples. Chromeleon 7.2 Data System Software (Thermo Scientific) was used to automate the measurements and process data.

Subsequently, we also measured substrate‐induced respiration as a proxy for soil microbial biomass (Anderson and Domsch 1978; Fierer, Schimel, 2003), although acknowledging that respiration may only be indicative of the active biomass. After the sampling of headspace gas for soil carbon mineralization, we added 4 ml of yeast extract solution (12 g yeast/L) to each tube, capped and flushed the tubes again with CO_2_‐free air and incubated them at 20°C for 4 hr. We then proceeded to sample headspace air and measure its CO_2_ concentration following the same protocol as for the soil carbon mineralization.

#### 2.3.5 Hydrolytic enzyme activity in the soil

At the end of each 6‐week phase, we measured potential activity of β‐glucosidase, acid phosphatase and alanine‐aminopeptidase enzymes using fluorometric assays (Baldrian 2009). We obtained soil homogenates by shaking (10 min, 330 rpm) 1 g of fresh soil suspended in 50 ml of sodium acetate buffer (2.5 mM, pH = 5.5). Fluorogenic substrates 4‐methylumbellyferyl‐β‐D‐glucopyranoside (MUFG), 4‐methylumbellyferyl‐phosphate (MUFP) and L‐alanine‐7‐amido‐4‐methylcoumarin (AMCA) were commercially obtained (Sigma‐Aldrich Chemie N.V.). We dissolved all substrates in dimethyl sulphoxide at concentrations of 2.5 mM for AMCA and 2.75 mM for MUFG and MUFP. A 40 µl of substrate solution was mixed with 250 µl of soil homogenate in each well of a black 96‐well plate. Three technical replicates were included per soil sample and enzyme activity. We calibrated concentrations of enzyme product with a dilution curve made from a stable form of the two fluorogenic compounds (1.0 mM methylumbellyferol and 1.0 mM 7‐aminomethyl‐4‐coumarin). We added 250 µl of soil homogenate to each well containing the 40 µl of the different dilutions to account for the potential influence of the soil homogenate on the fluorescence reading. Fluorescence was measured using a plate reader at the start of the incubation and after 2 hr of incubation at 40°C with an excitation and emission wavelengths of 360 and 460 nm, respectively (Synergy HT, BioTek Instruments). We compared the measured fluorescence in our samples, after subtraction of the blank, with the standard dilution curves to calculate the amount of enzymatic product formed over the incubation time. In our study, a unit of enzyme activity is defined as the amount of enzyme reaction product (µmol) per gram of dry soil per hour.

#### 2.3.6 Fungal community and relative abundance of saprophytic fungi

We identified the fungal composition and relative abundance of saprophytes in the soil samples at the end of drought, and after 6 and 12 weeks of recovery. We focused on the fungal community, because fungi are known to play a key role in decomposition processes (van der Wal, Geydan, Kuyper, & Boer, 2013). To determine fungal composition, we extracted DNA from soils samples stored at −80°C. Briefly, DNA was extracted from 0.25 g of soil using the PowerSoil DNA Isolation Kit (Mo Bio Laboratories) following the manufacturer's instructions. We then amplified DNA using duplicate PCRs with bar‐coded primers for multiplexing and re‐identifying individual samples following sequencing. Fungal community composition was determined by targeting the ITS2 region using ITS4 and ITS9 primers (Ihrmark et al., 2012). PCR products were purified using the Agencourt AMPure XP magnetic bead system (Beckman Coulter Life Sciences) with a volume ratio of PCR product to beads of 1:0.7. Purified PCR products were analysed in a fragment analyser using a Standard Sensitivity NGS Fragment Analysis Kit (1bp‐6000bp) and following manufacturer's instructions (Advanced Analytical Technologies GmbH). Finally, fungal ITS amplicons were sequenced using the Illumina MiSeq platform.

The ITS amplicon reads were analysed using the Hydra pipeline version 1.3.2 (de Hollander, 2017) implemented in Snakemake (Köster & Rahmann, 2012). Adapter sequences and PhiX contaminants were removed using BBDuk2 from the BBMap tool suite (Bushnell, 2015). Paired‐end reads were merged using the option fastq‐mergepairs of vsearch (Rognes, Flouri, Nichols, Quince, & Mahé, 2016). Sequences were converted to FASTA format and concatenated into a single file. All reads were clustered into OTUs using the UCLUST smallmem algorithm (Edgar, 2010). Chimeric sequences were detected using the UCHIME algorithm (Edgar, Haas, Clemente, Quince, & Knight, 2011). All reads were mapped to OTUs using the usearch‐global method implemented in VSEARCH, and a OTU Table was created and converted to BIOM format (McDonald et al., 2012). Taxonomic information for each OTU was obtained using the RDP Classifier re‐trained on the UNITE database 7.2 (Kõljalg et al., 2013). Soil samples with a read number lower than 1,000 reads and singletons were not included in further analyses. We obtained 1,147,313 reads of the ITS region. After removing singletons, 4,928 fungal OTUs were used in the analyses of community composition.

We analysed soil fungal community composition at OTU level and at functional level using the Funguild database (Nguyen et al., 2016). Fungal community samples were collected at the same times as the different soil functions were measured in our experiment (at the end of drought, 6 and 12 weeks after rewetting). We focused on the effect of the experimental treatments on fungal community composition and on the relative abundance of soil saprophytic fungi with respect to the total fungal community in order to link shifts in functional composition to differences in saprophytic activities measured. Thereby, we calculated the relative abundance of saprophytic fungi by accounting the total number of reads that were assigned to the saprophytic trophic mode (as a single trophic mode or in combination) within the total number of OTU reads in that sample (OTUs with other trophic modes and OTUs with no assigned guild).

### 2.4 Data analyses

We used general linear mixed effect models to test the effect of the experimental treatments on soil functions (litter mass loss, soil respiration, substrate‐induced respiration and enzyme activity), the relative abundance of soil fungi and plant shoot and root biomass. We included plant community origin, soil inoculum and drought as fixed factors and block as random factor. Satterthwaite approximation of denominator degrees of freedom was used with ‘lmerTest’ package in R (R Development Core Team, 2010). Enzyme activity rates, root biomass and relative abundance of saprophytic fungi data were log‐transformed when needed prior analyses to meet normality assumption in ANOVA as checked with Shapiro–Wilks test. Two extreme outlier data points of phosphatase and aminopeptidase activity were detected using the ‘outlier.test’ function in the ‘car’ package in R and removed from the analyses (R Development Core Team, 2010).

Subsequently, for each variable, we calculated the proportional change from the control as a measure of stability of the soil communities during drought (i.e. resistance) and at different stages after drought (i.e. resilience) (Griffiths et al., 2000). Negative values of this proportional change from the control indicate that drought negatively influenced that parameter with respect to the control. Positive values indicate that drought stimulated that parameter over the control levels. This relative difference to the control was always calculated within each of the five independent blocks. We then tested differences in stability (percentage of change from the control) between plant communities of different origin and soil inoculum using general linear mixed effect models, which did not include the fixed factor ‘drought’. All statistical tests were performed using R (R Development Core Team, 2010).

Canoco 5 software was used to conduct multivariate statistics on fungal community composition at OTU level (Ter Braak & Šmilauer, 2012). Relative abundances of fungal OTUs were log10‐transformed prior the analyses. We performed principal coordinate analyses (PCoA) of the dissimilarity matrix based on Bray–Curtis distances to visualize differences in fungal community composition. Thereafter, we statistically tested the effect of the experimental treatments on fungal community composition using PERMANOVA (999 permutations) (Oksanen et al., 2018). We carried out separate PERMANOVAs for each time period, with each of these analyses including all experiment variables (drought, soil inoculum, plant community).

## 3 RESULTS

### 3.1 Background environmental parameters

The soil moisture content at the start of the experiment was 20.8 ± 1.1%. At the end of the 6 weeks of drought, soil moisture in the drought treatment was reduced to 4.9 ± 0.6%, while it was 11.9 ± 1.6% in the control treatment. The drought treatment therefore induced a decrease in soil moisture of 58% of that in the control by the end of the drought event. After the 6‐week period of the early‐stage recovery, the soil moisture of the drought treatment levelled with that of the control (13.3 ± 1.2 and 13.6 ± 1.9%, respectively). At the end of the following six‐week period, late‐stage recovery, soil moisture was 16.1 ± 1.3% for the drought mesocosms and 16.4 ± 1.1% for the control.

Soil nitrate content was affected by plant community origin and soil inoculum before the drought started (Plant: *F*_1,32_ = 10.7, *p* = .002; Soil: *F*_1,32_ = 12.4, *p* = .001) and by soil inoculum only in the case of ammonium (Soil: *F*_1,32_ = 4.3, *p* = .044). Before the drought, soils had more nitrate in the presence of range‐expanding plant communities than in the presence of native communities, and northern soils had more soil nitrate and ammonium than southern soils.

At the end of the drought, soil nitrate content was negatively affected by drought under native plant communities with respect to the control, but not under range expanders (Soil × Plant: *F*_1,32_ = 17.1, *p* < .001), and in northern soils with respect to southern soils (Drought × Soil: *F*_1,32_ = 5.03, *p* = .031). The main effect of soil inoculum remained for ammonium, where the content was higher for northern soils compared to southern soils (Soil: *F*_1,32_ = 4.8, *p* = .034). Mesocosms subjected to drought had significantly higher content of ammonium than control mesocosms (Drought: *F*_1,32_ = 17.9, *p* < .001).

Neither above nor belowground plant biomass were affected by drought (shoot biomass: *F*_1,28_ = 0.26, *p* = .61; root biomass: *F*_1,28_ = 0.75, *p* = .39), plant community origin (shoot biomass: *F*_1,28_ = 0.16, *p* = .68; root biomass: *F*_1,28_ = 0.07, *p* = .79) or soil inoculum (shoot biomass: *F*_1,28_ = 2.10, *p* = .15; root biomass: *F*_1,28_ = 1.84, *p* = .18) (Figure S3).

### 3.2 Decomposition of high‐quality substrate

Litter mass loss was negatively affected by drought by an average of 12.9 ± 2.8% as measured at the end of the drought period (Figure 1a). During the early‐stage recovery, decomposition in the drought treatment recovered to control values (Figure 1b). Both during drought and early recovery, the proportional change from the control was not affected by plant community origin and soil inoculum (Table 1). However, after rewetting of the soil, mass loss of high‐quality litter tended to be higher in soils with a history of drought then in control soils (*p = .05*) (Tables S2 and S3). During the late recovery, there was a significant interaction between plant community origin and soil inoculum, where ‘home’ combinations (i.e. northern soils with native communities or southern soils with range‐expanding communities) had a positive proportional change, while this did not occur for ‘away’ combinations (i.e. northern soils with range‐expanding communities or southern soils with native communities combinations; Figure 1c, Table 1). In absolute terms, during the late recovery, litter mass loss was significantly higher in soils of native plant communities than in soils of range expanders (Tables S2 and S3).

**Table 1:** Linear mixed model output on the proportional changes from the control for all functional variables measured after the three phases of the experiment (drought, early and late recovery)

Fixed factors		Litter decomposition		Soil respiration		Enzyme activity			Saprophytic fungi
		High‐quality	Low‐quality	Soil respiration	Microbial biomass	β‐glucosidase	Phosphatase	Aminopeptidase	
During drought	Df	*F*_1,12_ (*p*‐value)	*F*_1,16_ (*p*‐value)	*F*_1,16_ (*p*‐value)	*F*_1,12_ (*p*‐value)	*F*_1,15_ (*p*‐value)	*F*_1,16_ (*p*‐value)	*F*_1,12_ (*p*‐value)	*F*_1,12_ (*p*‐value)
*Plant community (P)*	*1*	0.82 (ns)	1.838 (ns)	9.464 (**)	4.510 (.055)	3.7635 (.071)	0.8759 (ns)	5.7052 (*)	0.094 (ns)
*Soil inocula (S)*	*1*	0.061 (ns)	0.723 (ns)	0.636 (ns)	0.750 (ns)	8.2815 (**)	9.3049 (**)	5.1302 (*)	0.196 (ns)
*P x S*	*1*	3.782 (.075)	0.091 (ns)	4.416 (.051)	0.170 (ns)	0.3318 (ns)	0.0554 (ns)	0.0276 (ns)	0.467 (ns)

Early recovery	Df	*F*_1,16_ (*p*‐value)	*F*_1,12_ (*p*‐value)	*F*_1,16_ (*p*‐value)	*F*_1,12_ (*p*‐value)	*F*_1,15.99_ (*p*‐value)	*F*_1,15.99_ (*p*‐value)	*F*_1,11.41_ (*p*‐value)	*F*_1,11.99_ (*p*‐value)
*Plant community (P)*	*1*	1.158 (ns)	5.645 (*)	3.236 (.090)	5.534 (*)	0.1326 (ns)	0.5576 (ns)	0.1202 (ns)	4.117 (.06)
*Soil inocula (S)*	*1*	3.02 (ns)	1.082 (ns)	1.923 (ns)	0.085 (ns)	0.0561 (ns)	0.2199 (ns)	0.19939 (ns)	0.048 (ns)
*P x S*	*1*	3.669 (.073)	2.856 (ns)	0.971 (ns)	0.263 (ns)	0.0629 (ns)	0.4415 (ns)	0.13415 (ns)	0.000 (ns)

Late recovery	Df	*F*_1,12_ (*p*‐value)	*F*_1,16_ (*p*‐value)	*F*_1,16_ (*p*‐value)	*F*_1,16_ (*p*‐value)	*F*_1,15.99_ (*p*‐value)	*F*_1,14.99_ (*p*‐value)	*F*_1,12_ (*p*‐value)	*F*_1,8.7_ (*p*‐value)
*Plant community (P)*	*1*	0.008 (ns)	5.8 (*)	1.558 (ns)	1.605 (ns)	0.211 (ns)	0.8256 (ns)	0.02219 (ns)	3.385 (ns)
*Soil inocula (S)*	*1*	1.763 (ns)	1.756 (ns)	5.074 (*)	3.912 (.065)	1.2704 (ns)	3.4706 (.082)	0.05525 (ns)	0.262 (ns)
*P x S*	*1*	6.732 (*)	0.241 (ns)	3.247 (.090)	0.854 (ns)	0.5383 (ns)	0.3206 (ns)	0.6031 (ns)	3.265 (ns)

Note

Significance levels: ns *p* > .1; *p < .05; **p < .01; ***p < .001; ****p < .0001; *p*‐values between .1 and .05 are displayed.

### 3.3 Decomposition of low‐quality substrate

Mass loss of low‐quality litter was decreased by 26.2 ± 5.5% on average across all treatments with respect to the control during drought (Figure 1d), but there was no influence of plant community origin and soil inoculum on this reduction (Table 1). During both recovery phases, plant community origin significantly affected the proportional change from the control regardless of soil inoculum. Mass loss of low‐quality litter was more positively affected by drought history in soils under native plant communities than in soils under range expanders (Figure 1e,f, Table 1). There was no effect of soil inoculum on the change in litter mass loss during the recovery phases. Overall, litter mass loss was stimulated by drought history beneath native plant communities in both phases after rewetting, while it remained negatively affected beneath range‐expanding plant communities (Tables S2 and S3).

### 3.4 Soil basal respiration and substrate‐induced respiration (SIR)

**Figure 2:**
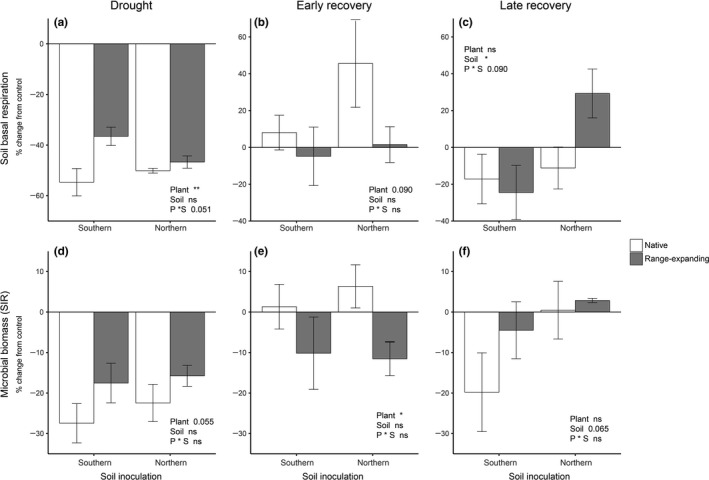
Proportional change from the control in soil basal respiration (a–c) and microbial biomass (d–f) at the end of the 6‐week experimental phases: drought (a, d), early recovery (b, e) and late recovery (c, f) after drought. Bars indicate means with standard error (*n* = 5)

Immediately after the drought period, soil respiration and SIR were more negatively affected by drought in soils under native plant communities than in soils under range‐expanding plant communities (Figure 2a,d, Table 1), indicating that in the presence of range‐expanding plant communities, soils are less negatively affected by drought than in the presence of native plants. Overall, drought decreased soil basal respiration and SIR in all soils (Tables S2 and S3). Soils of range expanders had higher SIR in southern inoculated soils than in northern inoculated soils and, overall, SIR was higher beneath native plant communities compared to range expanders at the end of the dry period (Tables S2 and S3). During the early recovery, SIR was more negatively affected by drought in soils under range‐expanding plant communities than in soils under natives (Figure 2b,e, Table 1). Although it was the same trend, this effect was not significant for soil respiration (Table 1). In the late recovery, the proportional change from the control in soil respiration and microbial biomass was negative for southern soils and positive for northern soils (Figure 2c,f, Table 1).

### 3.5 Enzyme activity

**Figure 3:**
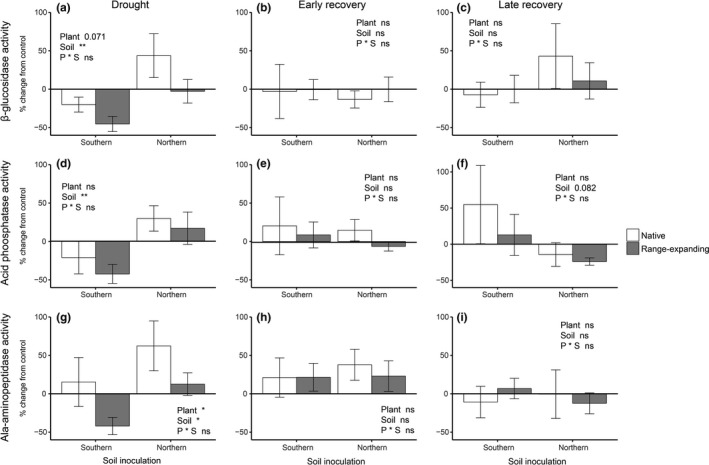
Proportional change from the control in b‐glucosidase (a–c), acid phosphatase (d–f) and alanine‐aminopeptidase (g–i) activities at the end of the 6‐week experimental phases: drought (a, d, g), early recovery (b, e, h) and late recovery (c, f, i) after drought. Bars indicate means with standard error (*n* = 5)

At the end of the drought period, the interaction between soil inoculum and drought affected the potential enzyme activity rates for all three enzymes. Drought stimulated glucosidase and phosphatase activity in northern soils but had decreased their activity in southern soils and vice versa for aminopeptidase activity (Tables S2 and S3). Drought also stimulated glucosidase activity beneath native plant communities but decreased it under range expanders. Overall, there was a positive effect of drought on enzyme activity in northern soils and a negative effect on southern soils at the end of the drought period (Figure 3a, Table 1). Glucosidase and phosphatase activity were higher in northern soils compared to southern soils during the recovery, but there was no significant effect of soil inoculum on aminopeptidase activity (Table S3). During both early and late recovery phases, there were no significant effects of plant community origin or soil inoculum on the proportional change from the control (Figure 3b,c, Table 1).

### 3.6 Saprophytic soil fungi and fungal community composition

**Figure 4:**
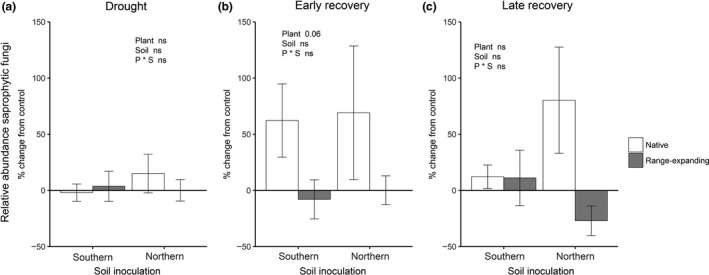
Proportional change from the control in the relative abundance of soil saprophytic fungi at the end of the 6‐week experimental phases: drought (a), early recovery (b) and late recovery (c) after drought. Bars indicate means with standard error (*n* = 5)

There were no differences in the relative abundance of saprophytic fungi between treatments at the end of the drought period (Tables S2 and S3). During early recovery, the relative abundance of soil saprophytic fungi tended to be higher in the presence of native plant communities with a drought history compared to the control (*p* = .08), but there was no effect of drought history for soils under range‐expanding communities (Tables S2 and S3). This effect was stronger during late recovery as was indicated by a significant plant community × drought interaction (Table S3). Saprophytic fungi in soils with drought were stimulated with respect to the control after rewetting of the soil, but only in soils under native plant communities and not in soils under range expanders (Figure 4, Table 1).

**Figure 5:**
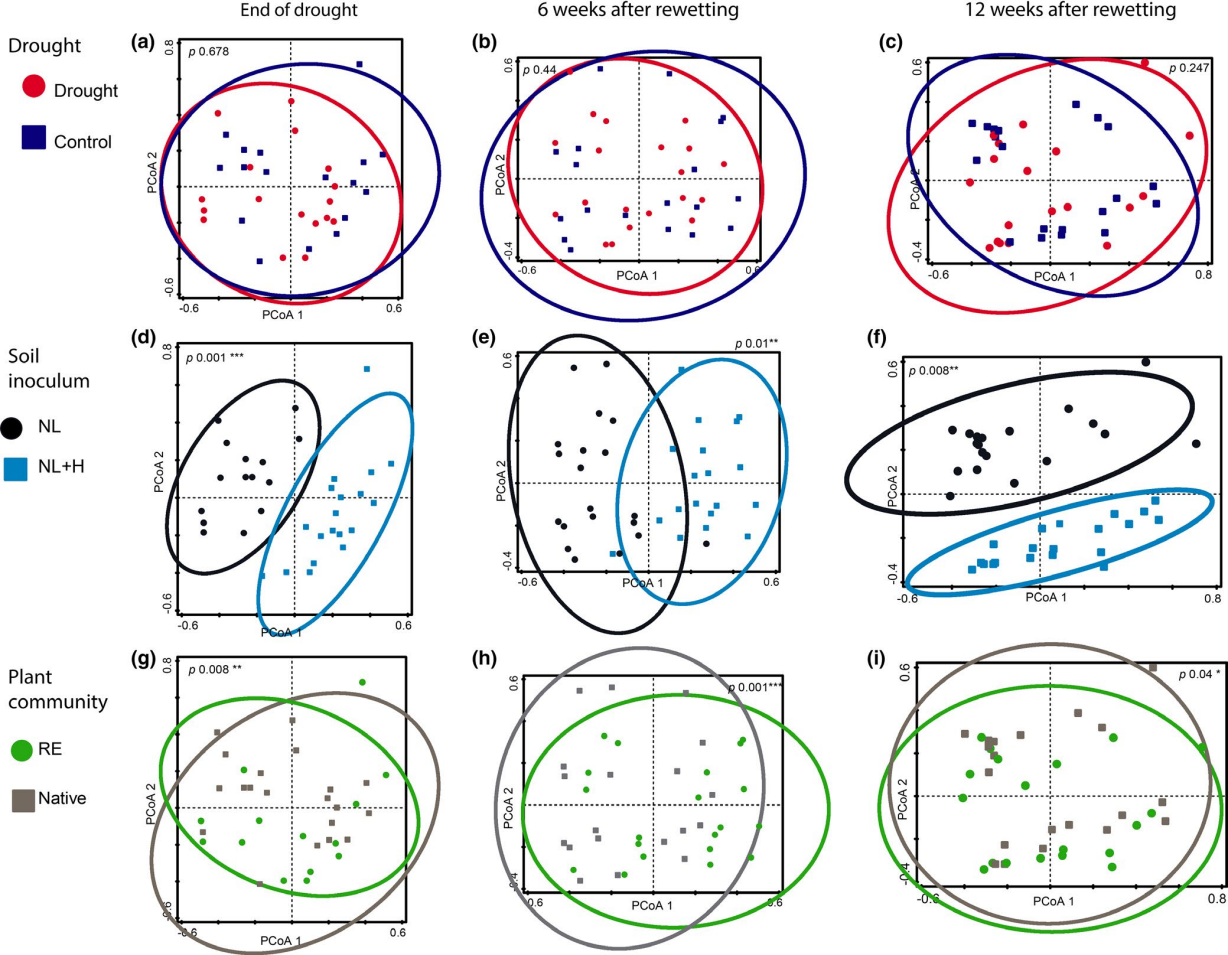
Principal coordinate analyses of the soil fungal community at the different experimental time points: after drought (a, d, g), six weeks (b, e, h) and twelve weeks after rewetting of the soil (c, f, i). We carried out separate PERMANOVAs for each time period, with each of the analyses including all experiment variables (drought, soil inoculum, plant community). For each experimental variable, we have created a separate plot to ease the interpretation of the data. The *p*‐values of the PERMANOVA tests are indicated in each panel

The composition of soil fungal communities differed between soil inoculation treatments prior to the drought treatment (Figure S2). The differences between northern and southern soils remain throughout the experiment (Figure 5d–f). Soil fungal communities were also significantly different between plant communities of native and range‐expanding plants (Figure 5g–i). In contrast, the experimental drought and subsequent soil rewetting had no effect on the composition of soil fungal communities (Figure 5a–c).

## 4 DISCUSSION

Multiple global change factors, such as species range shifts and extreme weather events, may affect both species composition in terrestrial ecosystems and important soil processes, such as litter decomposition and nutrient cycling. In order to predict how ecosystems will respond to global change, it is important to study both independent and interactive effects of the different components of global change. Here, we used a factorial experiment to test the hypotheses that soils under range‐expanding plants originating from south‐eastern Europe are more resistant and resilient to drought than soils under native plant communities, especially when range‐expanding plants grow in soils with south‐eastern soil biota. Our results show that plant community origin and soil inoculum can modify the magnitude and direction of the responses of soil functions to extreme drought. In general, responses were strongest shortly after rewetting and often associated with a positive effect of drought history on soil activity beneath native plant communities. However, these responses differed between specific soil functions measured and varied with the different experimental phases.

### 4.1 Responses to drought

In line with our first hypothesis, we found that soil respiration was less hampered by drought in soils under range expanders than in soils under native plant communities. Under moderate drought, plant species may increase root exudation and thereby maintain the activity of the soil microbial community in the root zone (Ahmed et al., 2018; Birgander, Rousk, & Olsson, 2017; Palta & Gregory, 1997; Preece & Peñuelas, 2016). However, we do not know whether plant origin in our study (native vs. range expander) resulted in different carbon inputs to the soil under drought. In contrast to our first hypothesis, plant community origin did not influence the resistance to drought for litter decomposition, substrate‐induced respiration (SIR) and the relative abundance of saprophytic fungi. Instead, in our study, the severe summer drought may have reduced soil community activity in both plant community treatments as a result of low substrate diffusion hampered by low soil water availability (Manzoni et al., 2012; Stark & Firestone, 1995).

In contrast to our expectation that soils with a southern inoculum would be more resistant to drought than soils with an inoculum from northern Europe (Hawkes et al., 2017), we found that both soils generally responded to drought in the same way. This means that within the latitudinal gradient selected, we found no evidence that differences in latitudinal origin of the soil influence current responses of soil community functioning to drought. Only for the response of enzyme activity did we find that soil inoculum played an important role. Interestingly, the interaction effect of soil inoculum and drought was dependent on the specific enzyme activity measured (e.g. phosphatase and glucosidase activity were enhanced by drought in northern soils, but decreased in southern soils). Even though the soil inocula only represented 20% of the topsoil in our mesocosms, differences in the soil mineral fraction between soil inoculum types may have influenced the stabilization of extracellular enzymes during periods of low water availability (Stursova & Sinsabaugh, 2008) and hence may explain differences in enzyme activities under drought. In addition, we found that the mineral N content in the soil at the start of our experiment depended on soil origin. As we did not measure mineral N content immediately upon soil inoculation, we cannot disentangle whether those differences are due to abiotic differences between the original inocula or weather differences were caused by the conditioning with plants during the experiment.

### 4.2 Responses to soil rewetting after drought

Our second hypothesis was that soil functions would recover faster from drought (i.e. higher resilience) in soils under range expanders, especially in combination with southern soil communities. However, we found rewetting to immediately stimulate decomposition of low‐quality substrate (i.e. rooibos tea), SIR and relative abundance of saprophytic fungi in soils under native plant communities, independent of soil inoculum. Soils from range‐expanding plants appeared to be more conservative in their response to drought. As a result, decomposition activity recovered more gradually and rewetting did not influence SIR and the relative abundance of saprophytic fungi in comparison to the control, suggesting limited effects of soil rewetting on microbial biomass and community composition in soils of range‐expanding plants.

It is well established that rewetting of dry soil generally enhances soil microbial activity (Birch, 1958). This so‐called Birch effect seems to be caused by metabolic adjustments at the level of individual microbial cells, which immobilize solutes intracellularly during drought and release them upon rewetting, thereby stimulating microbial activity (Fierer, Schimel, & Holden, 2003; Schimel, Balser, & Wallenstein, 2007). Our results show that native plants may have enhanced a potential Birch effect in the soil in terms of decomposition (low‐quality substrate) and SIR as compared to range expanders. Previous work has explained differences in the Birch effect between different plant communities via altered soil microbial communities (Fierer & Schimel, 2002). Our findings that SIR, as well as the relative abundance of saprophytic fungi increased under native communities, suggests that plant‐induced changes in the microbial biomass and community may indeed explain differences in the suggested Birch effect between native and range‐expanding plant communities. These plant‐induced effects may operate, for example, via drought‐induced root mortality and turnover or via different patterns of root exudation upon rewetting. Such effects, however, have not yet been shown for range‐expanding plant species.

In our study, we did not find an effect of drought history on soil respiration upon rewetting. Studies investigating responses of soil respiration after rewetting often focus on a time frame of hours to days after rewetting of the soil (Göransson, Godbold, Jones, & Rousk, 2013; Meisner, Leizeaga, Rousk, & Bååth, 2017). In contrast, we assessed the first responses to rewetting after 6 weeks, when immediate changes in soil respiration may have recovered already, while the impact of drought history on longer‐term processes, such as litter decomposition and changes in SIR, are still present. Therefore, responses of plants and soil communities to rewetting after drought may alter respiration rates immediately upon rewetting (Meisner, Bååth, & Rousk, 2013), promote changes on soil (saprophytic) microbial communities, decomposition and nutrient dynamics that persist to later stages after the drought stress (Fierer & Schimel, 2002; Fierer et al., 2003).

Overall, our experimental treatments had limited effects on soil processes and related processes did not always respond in the same way. For example, responses of soil respiration did not match those of litter mass loss. This may be explained by our sampling scheme, where some variables were measured at single time points (e.g. soil respiration and SIR), while others represent a cumulative process over the course of 6 weeks (e.g. litter mass loss). Furthermore, while a 6‐week drought period is a realistic climate scenario, the period of 6 weeks after rewetting may have been too long to capture the responses of soil respiration to rewetting, which often occur within a week (Fierer & Schimel, 2003; Göransson et al., 2013). Another aspect to take into account is the source of the seeds used. All seeds were sourced from the Netherlands (the expansion range), so that we do not have information on possible local adaptation between plant genotypes and the soil microbiome (Lau & Lennon, 2011). Further work is needed to obtain a better mechanistic understanding of these contrasting findings, for example by including more measurements on soil abiotic conditions, that may help to explain the responses of soil processes to drought under native and range‐expanding plant communities. Finally, our results suggested that process rates decreased over the recovery period. Although we cannot fully disentangle to what extent this is caused by removing the rain shelters after drought, a seasonal pattern may have been involved in this effect, as it turned fall already towards the end of the study period.

### 4.3 Plant response strategies

Our results that short‐term responses of soil functions to rewetting were dependent on plant community origin and soil inoculum indicate that plant range shifts can play a role in ecosystem responses to global change (Kardol et al., 2010). Even though climatic parameters can directly drive changes in soil communities and their functions, it has been shown that plants can modulate these effects via different mechanisms such as changes in soil microclimate, plant–soil interactions and the provisioning of carbon resources (Drigo et al., 2007; Dukes & Hungate, 2002; Waldrop & Firestone, 2006).

While the presence of native plant communities stimulated soil activity as a response to soil rewetting after drought, the presence of range‐expanding plants decreased the magnitude of the soil functioning responses to drought. Instead, soil functions beneath range‐expanding plants recovered to control levels without surpassing them, suggesting a more conservative response upon soil rewetting. This conservative response of soil functioning promoted by range‐expanding plants could be the result of adaptation to drought events, typically more incident in south and south‐eastern Europe where the range‐expanding plants originate from. In spite of the relatively limited nature of our experiment (i.e. single drought event, single year, short time frame), these results add to the discussion on whether range‐expanding plants may increase the stability of soil functions. In contrast to the effects of plant community origin, we found less impact of the origin of the soil inoculum on responses of ecosystem functions to drought, suggesting functional redundancy of soil communities from these different latitudes when compared under the same environmental conditions (Wertz et al., 2007).

Both the duration of drought periods and the frequency of drying–rewetting cycles can have important consequences for soil ecosystem functioning (Fierer & Schimel, 2002; Meisner, Bååth, et al., 2013). Short‐term dynamics of soil functions upon rewetting may become most relevant under a future climate scenario with recurrent drying and rewetting. Under such scenarios, plant range expansion may have the potential to substantially influence ecosystem‐level processes over longer time scales, such as net ecosystem productivity or soil carbon balance. Furthermore, as soil activity responses to drying–rewetting cycles can have a substantial contribution to global CO_2_ dynamics, especially in water‐limited ecosystems (Almagro, López, Querejeta, & Martínez‐Mena, 2009; Matteucci, Gruening, Goded Ballarin, Seufert, & Cescatti, 2015), we emphasize the need to also include the effect of changes in plant communities (e.g. as results of climate warming‐induced range shifts) on soil activity responses to drought. To understand the full potential of plant range expansion to modify soil ecosystem functioning in the longer term, it will be essential to study their interactive effects with future climate scenarios and to get a mechanistic understanding of these interactions.

## 5 CONCLUSIONS

We conclude that plant range expansion may influence short‐term responses of soil ecosystem processes to rewetting after drought periods and that these effects are independent of the geographical origin of the soil community. In particular, range‐expanding plant species from warm climate zones affected soil functioning in a more conservative manner following soil rewetting than native plant species. We propose that these different responses could have ecosystem‐level consequences depending on the nature and recurrence of the drought and on whether drought occurs during peak growing time for the plants or later in the season.

## AUTHORS' CONTRIBUTIONS

M.M., G.F.V. and W.H.P. designed the study. M.M., G.F.V., C.W., F.C.H., E.P.B., S.G. and K.S.R. performed the experiment and collected the data. H.M. performed molecular analyses. M.M. analysed the data with input from G.F.V. and W.H.P., M.M., G.F.V. and W.H.P. wrote the manuscript with input from all authors. Authors declare no conflict of interest. This is NIOO publication 6806.

## Data Availability

All soil, decomposition and plant biomass data have been deposited in the Dryad Digital Repository (https://doi.org/10.5061/dryad.7k6502b) (Manrubia, van der Putten, et al., 2019). Sequencing data have been submitted to the European Nucleotide Archive under the Accession Number PRJEB34236 (ERP117111).
