# Supplementary material for: Soil functional responses to drought under range‐expanding and native plant communities
Source: Funct Ecol. 2019 Sep 12;33(12):2402–16. doi: 10.1111/1365-2435.13453 (PMC6919305; doi:10.1111/1365-2435.13453)
Supplement: Supplementary file 1 [file FEC-33-2402-s001.pdf]

Functional Ecology

Soil functional responses to drought under range-expanding and native plant communities

M Manrubia, WH van der Putten, C Weser, FC ten Hooven, H Martens, EP Brinkman, S Geisen, KS Ramirez & GF Veen

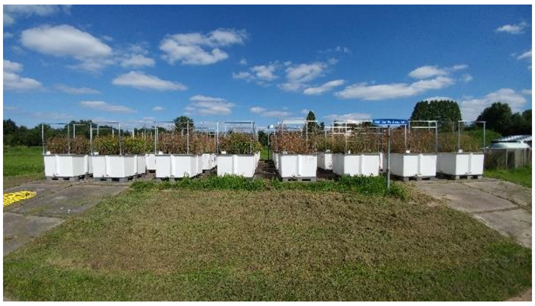

Climate change is affecting natural ecosystems in North-Western Europe in multiple ways. In the Netherlands, for example, climate predictions forecast an increase in extreme summer droughts. Simultaneously, plant species originating from Southern regions of Europe are becoming increasingly present in the Netherlands, facilitated by climate warming. Both drought and the arrival of novel plant species are known to control the processes that take place in the soil and that are of great importance for the functioning of natural ecosystems. However, we do not know how these two factors interact and modify soil processes in combination.

We investigated how ecosystems with local Dutch plants and ecosystems with novel plants from Southern Europe responded to drought. To do so, we assembled those ecosystems in big containers (mesocosms) and simulated a dry period. Then, we measured a series of soil functions such as plant litter decay and the activity of soil microbes.

We found that drought had negative impacts on all soil functions. However, after we added water to the dry soil again, we saw that the level of activity in soils of local Dutch plants was higher than that in soils of novel plants. Our study shows that the consequences of the different climate change factors (drought and plant species changes) are interacting with each other, and that their effects on the functions of soils are most important following the first rainfall after a dry period.
