# Supplementary material for: Soil functional responses to drought under range‐expanding and native plant communities
Source: Funct Ecol. 2019 Sep 12;33(12):2402–16. doi: 10.1111/1365-2435.13453 (PMC6919305; doi:10.1111/1365-2435.13453)

Supplementary Information

Soil functional responses to drought under range-expanding and native plant communities

M Manrubia[1], WH van der Putten[1,2], C Weser[1], FC ten Hooven[1], H Martens[3], EP Brinkman[1], S Geisen[1], KS Ramirez[1] & GF Veen[1]

[(1)] Department of Terrestrial Ecology, Netherlands Institute of Ecology (NIOO-KNAW), PO Box 50, 6700 AB, Wageningen, The Netherlands; [(2)] Laboratory of Nematology, Wageningen University, PO Box 8123, 6700 ES, Wageningen, The Netherlands; [(3)] Department of Soil Quality, Wageningen University, PO Box 47, 6700 AA, Wageningen, The Netherlands

Table S1. Native and range-expanding plant species planted in the mesocosms since its establishment in 2013. During the first 2 years of experiment (2013-2014), 3 range-expanding species and 3 related natives were planted in combination with 3 native grass species (*) to condition the soil. During 2015 and 2016, we planted the experimental plant communities. In 2016, the drought experiment was carried out.

**Plant community origin**	**2013-2014**	**2015 -2016**
**Native**	*Centaurea jacea* L.	*Centaurea jacea* L.
	Tragopogon pratensis L.	*Geranium molle* L.
	Angelica sylvestris L.	*Tragopogon pratensis* L.
		Rorippa sylvestris (L.)
	Phleum pratense L. *	*Sinapis arvensis* L.
	Poa trivialis L. *	*Crepis biennis* L.
	Festuca rubra L. *	*Pulicaria dysenterica* L. (Bernh.)
		Barbarea vulgaris (W.T. Aiton)
**Range expanding**	*Centaurea stoebe* L.	*Centaurea stoebe* L.
	Tragopogon dubius (Scop.)	*Geranium pyrenaicum* (Brum. F.)
	Angelica archangelica L.	*Tragopogon dubius* (Scop.)
		Rorippa austriaca (Crantz) Besser
	Phleum pratense L. *	*Rapistrum rugosum* (L.) All.
	Poa trivialis L. *	*Lactuca serriola* L.
	Festuca rubra L. *	*Dittrichia graveolens* (L.)
		Bunias orientalis L.
N^{o} individuals per species	6	8
Total number of plants	36	64

Table S2. All parameters measured at the end of each of the 3 phases of the experiment (during drought, early recovery and late recovery). RE indicates communities of range-expanding plant species. Substrate-induced respiration was used as a proxy for microbial biomass. Values are means ± standard error (n=5)

	Drought	Plant community	Soil inoculum	Litter decomposition (% of mass loss)		Soil respiration (µg CO_2-C * g soil^{-1} * h^{-1})		Enzyme activity (µmol * g soil^{-1} * h^{-1})			Saprophytic fungi
				High-quality	Low-quality	Soil respiration	Microbial biomass	*β*-glucosidase	Phosphatase	Aminopeptidase	(% of total fungi)
**During drought**	Control	Native	Northern	50.60 ± 1.6	19.19 ± 1.4	0.245 ± 0.02	0.982 ± 0.05	11.91 ± 2.0	11.20 ± 1.7	3.43 ± 0.5	25.11 ± 2.7
		Native	Southern	52.67 ± 1.0	18.45 ± 1.3	0.290 ± 0.04	1.089 ± 0.10	9.40 ± 1.6	11.60 ± 1.7	7.22 ± 1.2	24.50 ± 1.3
		RE	Northern	54.40 ± 2.1	19.97 ± 1.9	0.225 ± 0.02	0.930 ± 0.05	12.70 ± 1.4	10.45 ± 1.5	4.01 ± 0.6	24.48 ± 0.6
		RE	Southern	50.50 ± 1.6	17.75 ± 1.6	0.213 ± 0.02	0.906 ± 0.05	14.39 ± 1.7	14.82 ± 1.4	10.64 ± 1.4	25.71 ± 3.3
	Drought	Native	Northern	45.59 ± 1.7	14.65 ± 0.8	0.122 ± 0.01	0.753 ± 0.02	15.70 ± 1.9	13.81 ± 1.4	5.15 ± 0.8	27.75 ± 4.0
		Native	Southern	42.20 ± 3.0	16.10 ± 1.3	0.124 ± 0.01	0.772 ± 0.02	8.52 ± 1.2	8.34 ± 1.8	7.92 ± 2.6	24.01 ± 2.2
		RE	Northern	46.43 ± 2.9	12.08 ± 1.4	0.119 ± 0.01	0.780 ± 0.03	11.77 ± 1.4	11.00 ± 0.5	4.20 ± 0.4	23.11 ± 1.9
		RE	Southern	46.99 ± 1.1	12.81 ± 1.4	0.136 ± 0.01	0.739 ± 0.02	8.00 ± 1.7	8.70 ± 2.0	5.56 ± 0.5	28.17 ± 2.5
**Early recovery**	Control	Native	Northern	54.10 ± 2.3	24.68 ± 1.0	0.335 ± 0.03	1.273 ± 0.06	21.83 ± 2.0	16.42 ± 1.9	5.44 ± 0.8	24.34 ± 3.0
		Native	Southern	56.86 ± 1.3	22.82 ± 0.4	0.325 ± 0.02	1.262 ± 0.03	13.38 ± 1.9	11.65 ± 2.0	6.39 ± 0.8	23.16 ± 2.5
		RE	Northern	58.13 ± 1.1	21.83 ± 1.1	0.334 ± 0.02	1.408 ± 0.10	15.20 ± 1.1	14.03 ± 0.7	5.27 ± 0.8	25.23 ± 2.2
		RE	Southern	55.28 ± 1.3	25.54 ± 0.6	0.367 ± 0.07	1.342 ± 0.18	13.59 ± 2.6	12.22 ± 0.8	7.23 ± 1.4	24.94 ± 1.7
	Drought	Native	Northern	57.49 ± 1.9	24.29 ± 1.6	0.466 ± 0.05	1.343 ± 0.04	18.46 ± 2.1	17.98 ± 1.4	7.23 ± 1.1	35.94 ± 9.8
		Native	Southern	60.32 ± 2.1	26.73 ± 1.3	0.351 ± 0.04	1.276 ± 0.06	10.77 ± 3.8	11.44 ± 0.9	7.42 ± 1.5	34.77 ± 4.9
		RE	Northern	56.88 ± 1.5	20.25 ± 0.8	0.334 ± 0.03	1.230 ± 0.03	15.07 ± 2.6	13.15 ± 1.1	5.96 ± 0.3	24.65 ± 2.9
		RE	Southern	59.83 ± 2.0	22.99 ± 1.2	0.316 ± 0.03	1.143 ± 0.02	12.32 ± 0.6	12.81 ± 1.5	7.99 ± 1.0	21.87 ± 2.3
**Late recovery**	Control	Native	Northern	46.34 ± 2.5	11.24 ± 0.8	0.432 ± 0.05	1.208 ± 0.06	13.78 ± 2.3	16.02 ± 0.8	6.56 ± 1.0	20.68 ± 2.2
		Native	Southern	49.57 ± 2.1	11.85 ± 1.0	0.444 ± 0.05	1.483 ± 0.13	14.51 ± 2.2	11.47 ± 3.7	10.35 ± 2.6	26.50 ± 3.4
		RE	Northern	43.98 ± 2.6	13.23 ± 1.0	0.341 ± 0.03	1.140 ± 0.04	16.37 ± 1.4	20.78 ± 2.7	7.59 ± 1.4	31.82 ± 4.5
		RE	Southern	40.92 ± 1.9	12.21 ± 0.9	0.580 ± 0.17	1.372 ± 0.18	14.80 ± 2.5	12.18 ± 0.6	6.18 ± 1.0	24.82 ± 3.9
	Drought	Native	Northern	50.27 ± 1.7	14.49 ± 0.6	0.363 ± 0.02	1.200 ± 0.06	16.03 ± 0.8	13.71 ± 2.6	5.88 ± 1.8	36.67 ± 10.1
		Native	Southern	50.13 ± 1.9	12.78 ± 0.9	0.342 ± 0.02	1.143 ± 0.05	12.14 ± 1.6	12.15 ± 1.4	7.26 ± 0.7	28.31 ± 1.0
		RE	Northern	41.08 ± 1.6	12.77 ± 2.0	0.433 ± 0.04	1.172 ± 0.04	17.07 ± 2.2	15.34 ± 1.4	5.95 ± 0.6	22.02 ± 1.4
		RE	Southern	47.92 ± 2.7	10.86 ± 0.5	0.352 ± 0.02	1.273 ± 0.10	13.25 ± 1.7	13.58 ± 3.3	6.15 ± 0.6	23.86 ± 2.4

Table S3. Linear mixed model output for the different parameters measured after each of the three phases of the experiment (drought, early and late recovery)

		Litter decomposition		Soil respiration		Enzyme activity			Saprophytic fungi
Fixed factors		High-quality	Low-quality	Soil respiration	Microbial biomass	*β*-glucosidase	Phosphatase	Aminopeptidase	
**During drought**	Df	$F_{1,28}$ (p-value)	$F_{1,28}$ (p-value)	$F_{1,28}$ (p-value)	$F_{1,28}$ (p-value)	$F_{1,27.08}$ (p-value)	$F_{1,28}$ (p-value)	$F_{1,32}$ (p-value)	$F_{1,26}$ (p-value)
Plant community (P)	1	2.003 (ns)	2.119 (ns)	4.053 (*0.05*)	**4.360 (*)**	0.108 (ns)	0.000 (ns)	0.155 (ns)	0.000 (ns)
Soil inoculum (S)	1	0.835 (ns)	0.038 (ns)	1.355 (ns)	0.016 (ns)	**12.329 (***)**	0.745 (ns)	**26.750 (****)**	0.048 (ns)
Drought (D)	**1**	**27.702 (****)**	**24.645 (****)**	**113.353(****)**	**125.347 (****)**	2.889 (ns)	3.211(*0.08*)	0.166 (ns)	0.137 (ns)
P x S	1	0.154 (ns)	0.309 (ns)	0.899 (ns)	**4.243 (*)**	2.212 (ns)	**4.245(*)**	0.284 (ns)	1.497 (ns)
P x D	1	0.613 (ns)	2.236 (ns)	**5.620 (*)**	2.218 (ns)	**7.812 (**)**	2.011 (ns)	**4.314 (*)**	0.014 (ns)
S x D	1	0.039 (ns)	1.674 (ns)	0.100 (ns)	0.486 (ns)	**8.928 (**)**	**13.104(***)**	**6.694 (*)**	0.007 (ns)
P x S x D	1	3.752 (0.06)	0.037 (ns)	2.615 (ns)	0.214 (ns)	0.274 (ns)	0.052 (ns)	0.379 (ns)	0.638 (ns)
**Early recovery**	Df	$F_{1,28}$ (p-value)	$F_{1,32}$ (p-value)	$F_{1,32}$ (p-value)	$F_{1,32}$ (p-value)	$F_{1,28}$ (p-value)	$F_{1,28}$ (p-value)	$F_{1,32}$ (p-value)	$F_{1,31}$ (p-value)
Plant community (P)	1	0.072 (ns)	**6.133 (*)**	1.724 (ns)	1.373 (ns)	1.822 (ns)	1.973 (ns)	0.007 (ns)	1.596 (ns)
Soil inoculum (S)	1	1.297 (ns)	**4.840 (*)**	1.585 (ns)	3.614 (*0.06*)	**11.213 (**)**	**12.817 (***)**	3.399 (*0.07*)	0.003 (ns)
Drought (D)	1	4.154 (*0.05*)	0.035 (ns)	0.838 (ns)	1.224 (ns)	1.452 (ns)	0.079 (ns)	2.325 (ns)	0.981 (ns)
P x S	1	1.214 (ns)	3.376 (*0.07*)	0.632 (ns)	1.163 (ns)	3.699 (*0.06*)	**5.924 (*)**	1.313 (ns)	0.191 (ns)
P x D	1	0.505 (ns)	**5.747 (*)**	2.979 (*0.09*)	**4.584 (*)**	0.555 (ns)	0.186 (ns)	0.194 (ns)	3.157 (*0.08*)
S x D	1	1.383 (ns)	1.085 (ns)	1.693 (ns)	0.238 (ns)	0.003 (ns)	0.006 (ns)	0.033 (ns)	0.001 (ns)
P x S x D	1	1.314 (ns)	2.726 (ns)	0.373 (ns)	0.315 (ns)	0.095 (ns)	0.744 (ns)	0.053 (ns)	0.184 (ns)
**Late recovery**	Df	$F_{1,28}$ (p-value)	$F_{1,32}$ (p-value)	$F_{1,32}$ (p-value)	$F_{1,32}$ (p-value)	$F_{1,32}$ (p-value)	$F_{1,31}$ (p-value)	$F_{1,28}$ (p-value)	$F_{1,31}$ (p-value)
Plant community (P)	**1**	**14.777 (****)**	0.178 (ns)	0.039 (ns)	0.166 (ns)	0.864 (ns)	2.141 (ns)	1.205 (ns)	0.449 (ns)
Soil inoculum (S)	1	1.393 (ns)	1.731 (ns)	0.058 (ns)	2.516 (ns)	2.512 (ns)	**8.127 (**)**	1.088 (ns)	0.075 (ns)
Drought (D)	1	2.173 (ns)	0.606 (ns)	2.181 (ns)	1.359 (ns)	0.032 (ns)	0.473 (ns)	2.051 (ns)	0.341 (ns)
P x S	1	0.014 (ns)	0.351 (ns)	0.427 (ns)	0.192 (ns)	0.171 (ns)	0.118 (ns)	2.829 (ns)	0.589 (ns)
P x D	1	0.004 (ns)	3.803 (*0.05*)	1.574 (ns)	1.674 (ns)	0.018 (ns)	0.271 (ns)	0.305 (ns)	**5.603 (*)**
S x D	1	1.263 (ns)	1.090 (ns)	**4.969(*)**	2.532 (ns)	1.623 (ns)	2.610 (ns)	0.044 (ns)	0.020 (ns)
P x S x D	1	**5.187(*)**	0.214 (ns)	2.309 (ns)	0.830 (ns)	0.192 (ns)	0.061 (ns)	1.116 (ns)	3.352 (*0.07*)

Significance levels: ns p > 0.1; * p<0.05; ** p<0.01; *** p<0.001; **** p<0.0001; p-values between 0.1 and 0.05 are displayed

Figure S1. Mesocosms after the installation of the rain shelters. The 40 mesocosms were distributed in 5 rows of 8 units. Each row contained an experimental block (replicate) and treatments within the blocks were assigned in a randomized way.

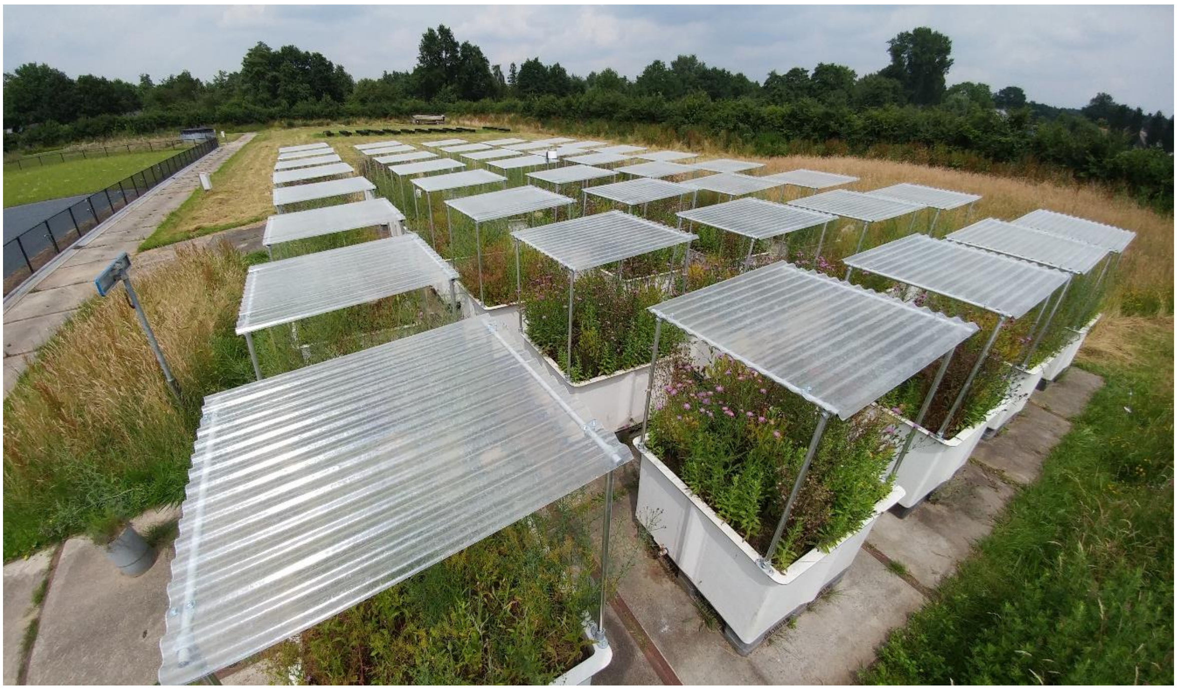

Figure S2. Principal Coordinate Analyses of the soil fungal community prior the drought event. Soils that received northern soil inoculum are represented in black circles and soils that received southern soil inoculum are represented in blue squares. Statistical significance and r^2 of the PERMANOVA test (999 permutations) to determine the effect of soil inoculation on fungal communities are indicated.

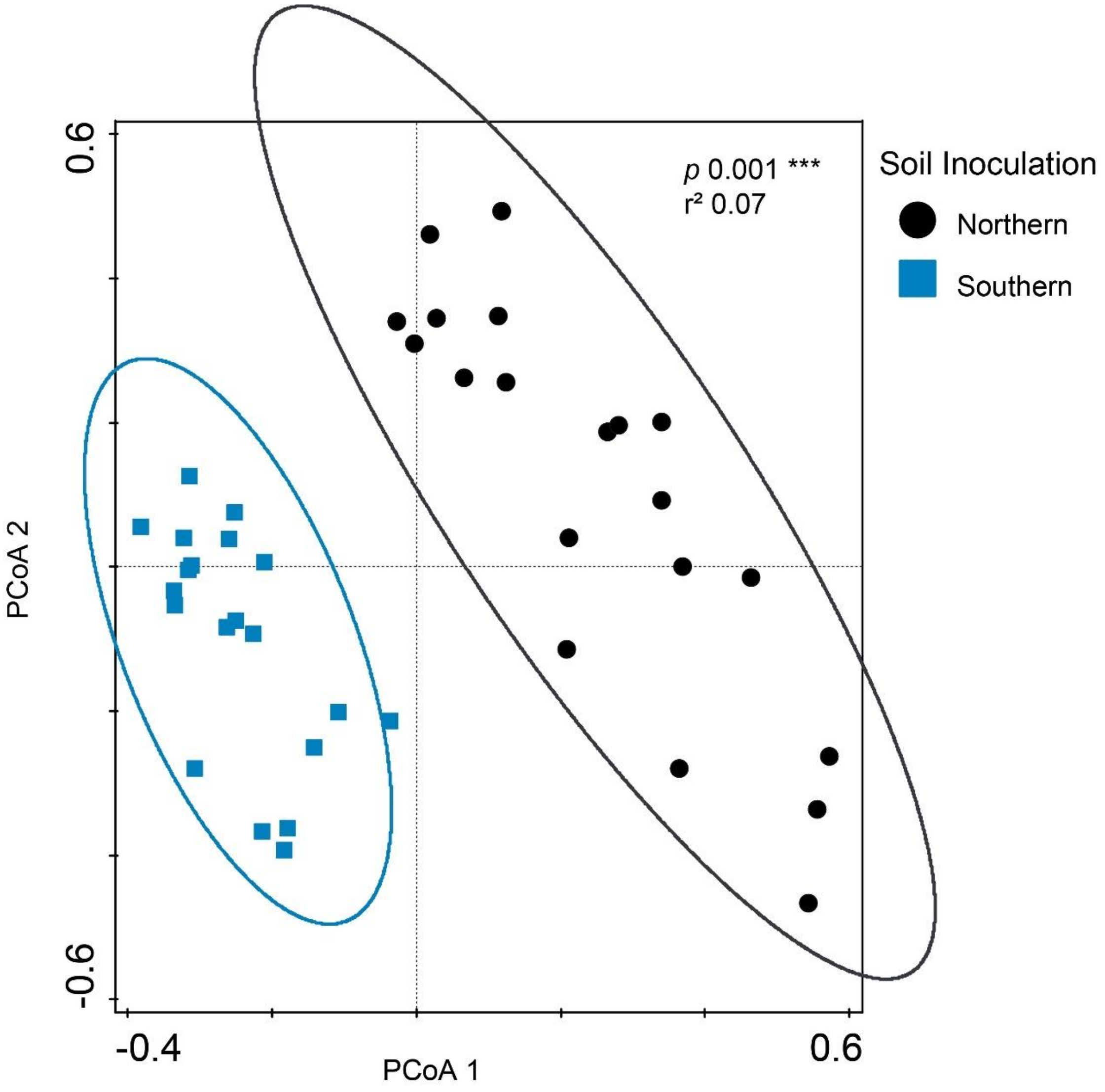

Figure S3. Shoot and root biomass at the end of the growing season. Bars indicate means with standard error (n=5).

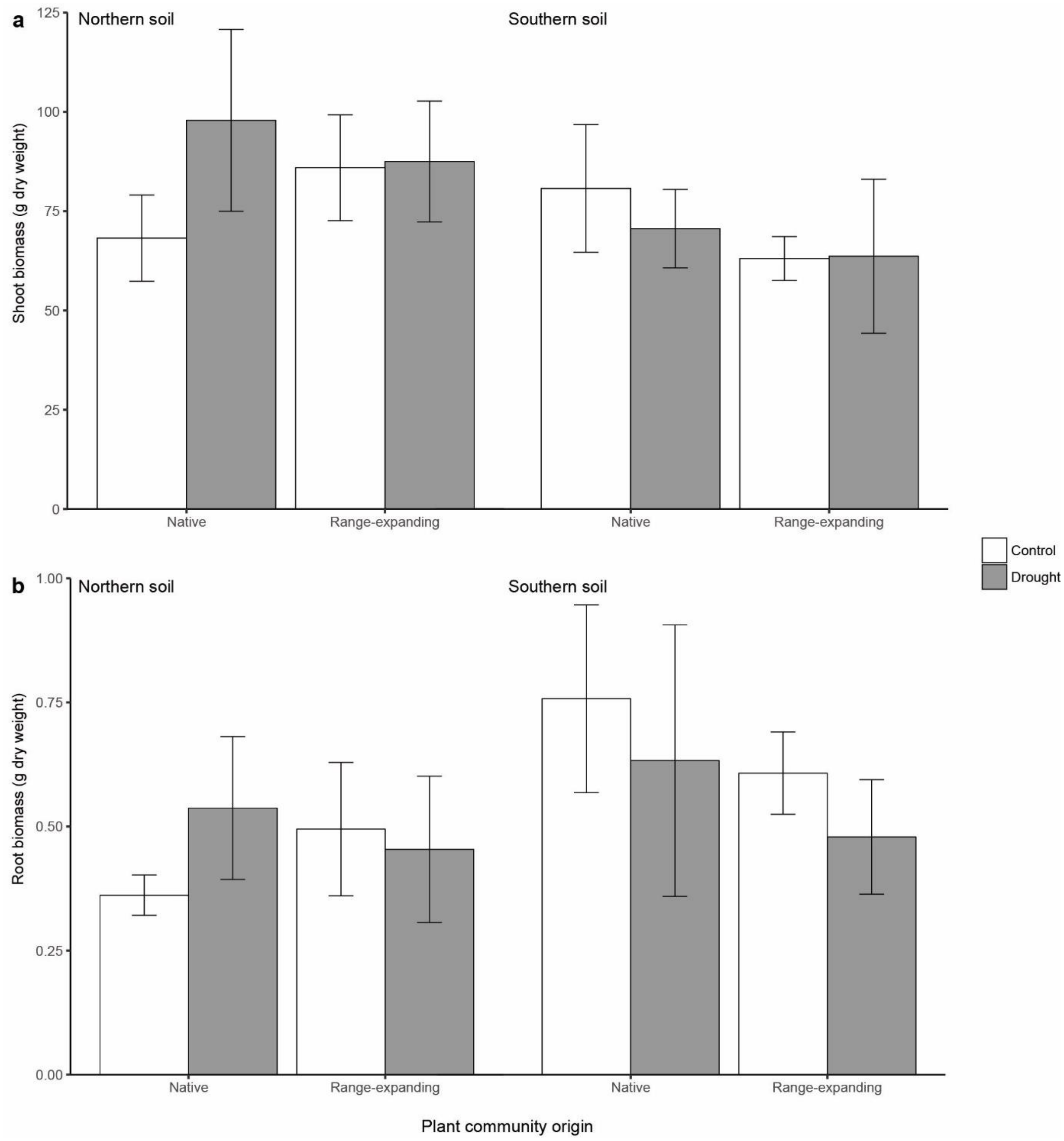

Supplement: Supplementary file 2 [file FEC-33-2402-s002.pdf]
